# 123 Sleep Disorder Is Associated with Neuropsychological Disturbances in Burn Survivals

**DOI:** 10.1093/jbcr/irac012.125

**Published:** 2022-03-23

**Authors:** Jasmine M Chaij, George Golovko, Juquan Song, Steven E Wolf, Amina El Ayadi, Kendall Wermine, Sunny Gotewal, Lyndon G Huang, Giovanna De La Tejera, Elvia L Villarreal, Kassandra K Corona, Tsola A Efejuku, Phillip H Keys

**Affiliations:** University of Texas Medical Branch, Galveston, Texas; University of Texas Medical Branch at Galveston, Galveston, Texas; University of Texas Medical Branch, Galveston, Texas; University of Texas Medical Branch at Galveston, Galveston, Texas; University of Texas Medical Branch at Galveston, Galveston, Texas; University of Texas Medical Branch, Galveston, Texas; University of Texas Medical Branch, Galveston, Texas; University of Texas Medical Branch, Galveston, Texas; University of Texas Medical Branch, Galveston, Texas; University of Texas Medical Branch, Galveston, Texas; University of Texas Medical Branch, Galveston, Texas; University of Texas Medical Branch, Galveston, Texas; University of Texas Medical Branch, Galveston, Texas

## Abstract

**Introduction:**

Prior studies explored neuropsychological disorders in the context of burn severity; however, the relationship between occurrence after burn and sleep has not been investigated. This study aims to determine if patients that developed a first-time sleep disorder after burn injury are more likely to develop a psychological or nervous system disorder within 10 years after injury.

**Methods:**

We identified burn patients on the TriNetX database, a federated research network of de-identified patient data. We formed two groups, those with first time sleep disorder diagnosis on or after the incidence of burn injury and those with no first-time sleep disorder diagnosis on or after burn. Groups were propensity matched to evaluate incidence of nervous system and mental disorders and characteristics, defined as bipolar disorder, epilepsy, neuropathy disorders, and 52 other neuropsychological disorders. Diagnoses of nervous system disorder and mental disorder were limited to after the burn injury and within the 10-year time frame. We analyzed data using a z-test with a p < 0.05 considered significant.

**Results:**

We found 7.83% of patients developed a first-time sleep disorder after burn injury. The population was older (43.9 ± 20.8 vs. 31.7 ± 22.4 yrs), female (51.13% vs. 46.10%), and White (70.02% vs 60.24%) when compared to those without sleep disorders (p< 0.05). Those who experienced a first-time sleep disorder after burn presented a greater risk of developing the mental, central nervous system, and peripheral nervous system disorders when compared to those who did not. Eating disorders, persistent mood disorders, and obsessive-compulsive disorders were 4.54, 95% CI [3.65, 5.65]; 3.84, 95% CI [3.49, 4.22], and 3.94, 95% CI [3.13, 4.97] times higher, respectively, in patients who developed a first-time sleep disorder (p< 0.05). Anxiety-related disorders were also more than 3 times more likely in those who developed a sleep disorder after burn (p< 0.05).Central nervous system disorders were related to sleep disorder post burn. Extrapyramidal and movement disorders were more than 3 times more likely to occur in sleep disorder patients (Extrapyramidal and movement disorder, unspecified 95% CI [2.48, 4.63] and Other extrapyramidal and movement disorders 95% CI [3.17, 3.78]. In regard to peripheral nervous system disturbances, restless leg syndrome was more than 4 times more likely to occur in patients that developed a first time sleep disorder after burn injury 95% CI [3.70, 4.65]. Polyneuropathy was also 2.28 more times likely to occur 95% CI [2.12, 2.47].

**Conclusions:**

Mental disorders and various central nervous system and peripheral nervous system disturbances are highly associated with identification of sleep disorders after burn. This finding suggests close monitoring for sleep in those who were burned to optimize outcomes.

